# Factors Influencing the Utilization of Diabetes Complication Tests Under the COVID-19 Pandemic: Machine Learning Approach

**DOI:** 10.3389/fendo.2022.925844

**Published:** 2022-06-22

**Authors:** Haewon Byeon

**Affiliations:** ^1^Department of Digital Anti-aging Healthcare (BK21), Graduate School of Inje University, Gimhae, South Korea; ^2^Department of Medical Big Data, College of AI Convergence, Inje University, Gimhae, South Korea

**Keywords:** complication test, diabetes, fundus examination, microprotein urination test, COVID-19 pandemic

## Abstract

**Objective:**

There are still not enough studies on the prediction of non-utilization of a complication test or a glycated hemoglobin test for preventing diabetes complications by using large-scale community-based big data. This study identified the ratio of not taking a diabetes complication test (fundus examination and microprotein urination test) among adult diabetic patients over 19 years using a national survey conducted in South Korea and developed a model for predicting the probability of not taking a diabetes complication test based on it.

**Methods:**

This study analyzed 25,811 subjects who responded that they had been diagnosed with diabetes by a doctor in the 2020 Community Health Survey. Outcome variables were defined as the utilization of the microprotein urination test and the fundus examination during the past year. This study developed a model for predicting the utilization of a diabetes complication test using logistic regression analysis and nomogram to understand the relationship of predictive factors on the utilization of a diabetes complication test.

**Results:**

The results of this study confirmed that age, education level, the recognition of own blood glucose level, current diabetes treatment, diabetes management education, not conducting the glycated hemoglobin test in the past year, smoking, single-person household, subjectively good health, and living in the rural area were independently related to the non-utilization of diabetes complication test after the COVID-19 pandemic.

**Conclusion:**

Additional longitudinal studies are required to confirm the causality of the non-utilization of diabetes complication screening tests.

## Introduction

The International Diabetes Federation (2019) (1) estimated that 463 million people, or 1 out of 11 adults (9.3%), worldwide had diabetes as of 2019. Especially, as of 2019, 136 million older adults (≥65 years old), one out of five older adults, suffered from diabetes (1). It is predicted that the number of actual diabetic patients can be higher than the estimated number when including potential diabetic patients who are not diagnosed with diabetes, yet suffering from severe diabetes symptoms. It is noteworthy that the prevalence of diabetes is increasing rapidly every year in these statistics. It has been forecasted that, if this trend continues, the number of diabetic patients worldwide will be 578 million (10.2%) in 2030 and 700 million (10.9%) in 2045, a 51% increase from 2019 (1).

It is believed that complications related to diabetes (e.g., microvascular complications and macrovascular complications) will increase rapidly along with the increase in the prevalence of diabetes. Numerous studies (2–5) have scientifically proven that thorough blood glucose regulation can reduce the risk of diabetes complications. Moreover, the Korean Diabetes Association recommends strictly controlling the blood glucose of diabetic patients by distributing the Diabetes Management Guidelines to diabetic patients and high diabetes risk groups (e.g., impaired glucose tolerance and impaired fasting glucose) living in local communities for the past 20 years (6, 7). In particular, the Diabetes Management Guidelines (2019) (7) recommends receiving the glycated hemoglobin test once every three months, and kidney tests and eye disease test at least once a year.

However, these recommendations are not well followed by diabetic patients living in local communities. It has been reported that 60% of diabetic patients in South Korea do not meet the blood glucose regulation goal (8) and only 25% of diabetic patients take tests for screening diabetic complications (e.g., microalbuminuria test and funduscopy) (8, 9). It is necessary to understand the characteristics of diabetic patients in South Korea participating in the examination for diabetes complications and prepare measures to improve the participation of those who have not taken these tests in order to prevent complications of diabetes and to properly manage diabetes.

Many previous studies (10–13) indicated the disease duration of diabetes, age, education level, residential area, income level, diabetes education, and subjective health status were factors associated with the fundus examination of diabetic patients. Moreover, other studies reported that sex, age, hospitalization experience, and comorbidities were related to receiving using the glycated hemoglobin test (14, 15). However, there are still not enough studies on the prediction of non-utilization of a complication test or a glycated hemoglobin test for preventing diabetes complications by using large-scale community-based big data.

Particularly, the importance of managing diabetic patients’ health at home is increasing due to the extended COVID-19 pandemic that started in November 2019 and preventive measures against it such as social distancing. This study identified the ratio of not taking a diabetes complication test among adult diabetic patients over 19 years using a national survey conducted in South Korea and developed a model for predicting the probability of not taking a diabetes complication test based on it.

## Materials and Methods

### Subjects

This study is an epidemiological study using secondary data based on the 2020 Community Health Survey. The Community Health Survey is a survey conducted under the supervision of the Korea Disease Control and Prevention Agency to produce health statistics necessary for establishing local health and medical plans and performing health services. The 2020 Community Health Survey extracted sample locations from the sampling frame, which was generated by linking the housing data and resident registration demographic data. They were complete enumeration surveys and they targeted adults (19 years or older) based on the resident registration in all municipalities (i.e., cities, guns, and gus) in South Korea. These sampling locations were allocated to each region (dong, eup, and myeon). Sample households were selected using a systematic sampling method by identifying the number of households chosen as sample points. This study collected data using the computer assisted personal interviewing method, in which a trained investigator conducted a 1:1 interview with a subject using a laptop computer. Finally, this study analyzed 25,811 subjects among 26,839 people who responded that they had been diagnosed with diabetes by a doctor in the 2020 Community Health Survey after excluding 1,028 who had answered items related to the utilization of the microprotein urination test and fundus examination insufficiently.

### Measurement

Outcome variables were defined as the utilization of the microprotein urination test and the fundus examination during the past year (yes or no). The utilization of diabetic eye complication examination was defined by the positive answer (“yes”) to the question (“Have you ever received a fundus examination to find whether you have an eye complication due to diabetes in the past year?”). The utilization of diabetic kidney complication examination was defined by the positive answer (“yes”) to the question (“Have you ever received a microprotein urination test other than the urine test stick to find whether you have a kidney complication due to diabetes in the past year?”).

By referring to Andersen’s health service utilization model (16), explanatory variables included gender, age (≤49, 50-59, 60-69, 70-79, or ≥80), education level (elementary school graduation and below, middle school graduation, high school graduation, or college graduation or above), household composition (single-person household, two-person household, or household with three or more members), marital status (with a spouse, single/divorced, or without a spouse due to widowed/separated), mean monthly household income (≤1.99 million KRW, 2.00-3.99 million KRW, or ≥4 million KRW), economic activity (yes or no) (17), residential area (urban or rural), smoking (yes or no), drinking (yes or no), regular exercise for more than 5 days or more and 30 minutes per time (yes or no), the recognition of own blood glucose level (yes or no), the recognition of own blood pressure level (yes or no), the application of diabetes treatment (oral hypoglycemic agent, insulin injection, and non-drug treatment) (yes or no), the number of glycated hemoglobin tests in the past year (do not know what glycated hemoglobin is, not measured, 1 time, 2 times, 3 times, or 4 times or more), the completion of diabetes management education (yes or no), and subjective health status (good, moderate, or poor).

### Development of Predictive Models

This study developed a model for predicting the utilization of a diabetes complication test (kidney disease and eye disease) using logistic regression analysis to understand the relationship of predictive factors on the utilization of a diabetes complication test. The backward selection method was used to select variables in the regression model. This study presented an adjusted odds ratio (AOR) and 95% confidence interval (CI) by developing an adjusted multivariate model that corrected all confounding factors.

The developed model (multivariate model) for predicting diabetes complications test was converted into a nomogram so that medical workers could easily interpret the predictive probabilities of high-risk groups. The nomogram developed in this study consisted of four elements. First, the point line is a line placed at the top of the nomogram to derive a score corresponding to a category of risk factors. It is between 0 and 100 points in the logistic nomogram. Second, the number of risk factor lines was as many as the number of risk factors. Third, the total point line is the sum of all individual risk factors and is at the bottom of the nomogram. Fourth, the probability line was placed at the bottom of the nomogram to derive the non-utilization probability of the diabetes complication test. This study presented only the top ten variables with high weights for the nomogram to make interpretation easier.

This study used 10-fold cross-validation to test the accuracy of the developed predicting the utilization of a diabetes complication test nomogram to minimize the risk of overfitting. This study presented the area under the curve (AUC), general accuracy, precision, recall, and calibration plot. AUC refers to the area under the receiver operating characteristic (ROC) curve. It is the most commonly used evaluation method in binary classification. It is defined as diagnostic accuracy. When the value is closer to 1, the diagnostic performance gets higher. A calibration plot is to visually confirm the degree of agreement between the prediction probability and the actually observed probability in the nomogram.

## Results

### Characteristics of Subjects According to the Utilization of Complication Examinations in South Korean Diabetic Patients After the COVID-19 Pandemic

**Tables 1**, **2** show the characteristics of subjects according to the utilization of complication tests (kidney disease and eye disease) in South Korean diabetic patients. The results of chi-square test showed that the utilization of diabetic eye disease complication test was significantly affected by the recognition of own blood pressure level, the recognition of own blood glucose level, non-drug treatment for diabetes, oral diabetes medication, diabetes insulin treatment, diabetes management education, the completion of diabetes management education, the number of glycated hemoglobin tests in the past year, age, household composition, economic activity, education level, marital status, subjective health status, smoking, binge drinking in the past year, and residential area (p<0.05).

**Table 1:** Characteristics of subjects according to the diabetic eye complication test (fundoscopic examination), n (%).

Factors	Diabetic eye complication test		p
	Yes (n=10,480)	No (n=15,331)	
Recognition of own blood pressure level			<0.001
Yes	7,467 (44.7)	9,233 (55.3)	
No	3,002 (33.1)	6,057 (66.9)	
Recognition of own blood glucose level			<0.001
Yes	7,700 (45.5)	9,224 (54.5)	
No	2,765 (31.3)	6,067 (68.7)	
Non-drug treatment for diabetes			<0.001
Yes	4,108 (48.8)	4,318 (51.2)	
No	6,368 (36.6)	11,012 (63.4)	
Oral diabetes medication			<0.001
Yes	9,995 (41.5)	14,115 (58.5)	
No	481 (28.4)	1,214 (71.6)	
Diabetes insulin treatment			<0.001
Yes	1,294 (68.8)	587 (31.2)	
No	9,185 (38.4)	14,743 (61.6)	
Completion of diabetes management education			<0.001
Yes	3,072 (59.7)	2,071 (40.3)	
No	7,405 (35.8)	13,255 (64.2)	
Number of glycated hemoglobin tests in the past year			<0.001
Do not know what glycated hemoglobin is	1,942 (27.7)	5,075 (72.3)	
4 times or more	3,632 (57.8)	2,653 (42.2)	
3 times	1,327 (51.9)	1,232 (48.1)	
2 times	1,776 (47.5)	1,962 (52.5)	
1 time	1,103 (41.0)	1,586 (59.0)	
Not measured	654 (19.3)	2,728 (80.7)	
Age			<0.001
≤49	609 (40.0)	913 (60.0)	
50-59	1,910 (42.2)	2,612 (57.8)	
60-69	3,445 (43.0)	4,573 (57.0)	
70-79	3,389 (40.8)	4,908 (59.2)	
≥80	1,127 (32.6)	2,325 (67.4)	
Household composition			<0.001
Single-person household	2,020 (36.3)	3,546 (63.7)	
Two-person household	5,561 (41.0)	7,998 (59.0)	
Household with three or more members	2,899 (43.4)	3,787 (56.6)	
Economic activity			<0.001
Yes	4,889 (38.7)	7,757 (61.3)	
No	5,583 (42.4)	7,569 (57.6)	
Education level			<0.001
Elementary school graduation and below	3,911 (35.2)	7,185 (64.8)	
Middle school graduation	1,906 (41.0)	2,739 (59.0)	
High school graduation	2,949 (45.1)	3,588 (54.9)	
College graduation or above	1,706 (48.7)	1,799 (51.3)	
Marital status			<0.001
Married	7,525 (41.9)	10,419 (58.1)	
Single	230 (39.7)	350 (60.3)	
Divorced, widowed, or separated	2,722 (37.4)	4,548 (62.6)	
Household income			<0.001
<2 million KRW	4,308 (37.6)	7,159 (62.4)	
2-3 million KRW	1,517 (43.2)	1,991 (56.8)	
3-4 million KRW	1,074 (43.0)	1,424 (57.0)	
>4 million KRW	2,067 (46.4)	2,392 (53.6)	
Subjective health status			<0.001
Good	1,371 (38.1)	2,230 (61.9)	
Moderate	3,923 (38.7)	6,220 (61.3)	
Poor	5,186 (43.0)	6,879 (57.0)	
Regular moderate level exercise			0.315
No	8,983 (40.5)	13,206 (59.5)	
Yes	1,491 (41.4)	2,113 (58.6)	
Smoking			<0.001
Non-smoker	5,979 (41.2)	8,530 (58.8)	
Ex-smoker	3,040 (41.5)	4,287 (58.5)	
Current smoker	1,461 (36.8)	2,514 (63.2)	
Binge drinking in the past year			<0.001
No	9,205 (41.2)	13,119 (58.8)	
Yes	1,273 (36.6)	2,209 (63.4)	
Gender			0.317
Male	5,083 (40.3)	7,533 (59.7)	
Female	5,397 (40.9)	7,798 (59.1)	
Residential area			<0.001
Urban	5,772 (47.2)	6,458 (52.8)	
Rural	4,708 (34.7)	8,873 (65.3)	

**Table 2:** Characteristics of subjects according to the diabetic kidney complication test (microprotein urination test), n (%).

Factors	Diabetic kidney complication test		p
	Yes (n=12,439)	No (n=13,372)	
Recognition of own blood pressure level			<0.001
Yes	8,801 (52.7)	7,899 (47.3)	
No	3,621 (40.0)	5,438 (60.0)	
Recognition of own blood glucose level			<0.001
Yes	9,058 (53.5)	7,866 (46.5)	
No	3,362 (38.1)	5,470 (61.9)	
Non-drug treatment for diabetes			<0.001
Yes	4,830 (57.3)	3,596 (42.7)	
No	7,608 (43.8)	9,772 (56.2)	
Oral diabetes medication			<0.001
Yes	11,865 (49.2)	12,245 (50.8)	
No	571 (33.7)	1,124 (66.3)	
Diabetes insulin treatment			<0.001
Yes	1,364 (72.5)	517 (27.5)	
No	11,074 (46.3)	12,854 (53.7)	
Completion of diabetes management education			<0.001
Yes	3,409 (66.3)	1,734 (33.7)	
No	9,027 (43.7)	11,633 (56.3)	
Number of glycated hemoglobin tests in the past year			<0.001
Do not know what glycated hemoglobin is	2,200 (31.4)	4,817 (68.6)	
4 times or more	4,207 (66.9)	2,078 (33.1)	
3 times	1,603 (62.6)	956 (37.4)	
2 times	2,250 (60.2)	1,488 (39.8)	
1 time	1,391 (51.7)	1,298 (48.3)	
Not measured	732 (21.6)	2,650 (78.4)	
Age			<0.001
≤49	798 (52.4)	724 (47.6)	
50-59	2,446 (54.1)	2,076 (45.9)	
60-69	3,979 (49.6)	4,039 (50.4)	
70-79	3,886 (46.8)	4,411 (53.2)	
≥80	1,330 (38.5)	2,122 (61.5)	
Household composition			<0.001
Single-person household	2,328 (41.8)	3,238 (58.2)	
Two-person household	6,567 (48.4)	6,992 (51.6)	
Household with three or more members	3,544 (53.0)	3,142 (47.0)	
Economic activity			0.002
Yes	5,968 (47.2)	6,678 (52.8)	
No	6,463 (49.1)	6,689 (50.9)	
Education level			<0.001
Elementary school graduation and below	4,623 (41.7)	6,473 (58.3)	
Middle school graduation	2,258 (48.6)	2,387 (51.4)	
High school graduation	3,535 (54.1)	3,002 (45.9)	
College graduation or above	2,009 (57.3)	1,496 (42.7)	
Marital status			<0.001
Married	9,010 (50.2)	8,934 (49.8)	
Single	261 (45.0)	319 (55.0)	
Divorced, widowed, or separated	3,160 (43.5)	4,110 (56.5)	
Household income			<0.001
<2 million KRW	5,031 (43.9)	6,436 (56.1)	
2-3 million KRW	1,793 (51.1)	1,715 (48.9)	
3-4 million KRW	1,348 (54.0)	1,150 (46.0)	
>4 million KRW	2,483 (55.7)	1,976 (44.3)	
Subjective health status			0.006
Good	1,667 (46.3)	1,934 (53.7)	
Moderate	4,844 (47.8)	5,299 (52.2)	
Poor	5,928 (49.1)	6,137 (50.9)	
Regular moderate level exercise			0.003
No	10,607 (47.8)	11,582 (52.2)	
Yes	1,820 (50.5)	1,784 (49.5)	
Smoking			0.022
Non-smoker	6,890 (47.5)	7,619 (52.5)	
Ex-smoker	3,623 (49.4)	3,704 (50.6)	
Current smoker	1,926 (48.5)	2,049 (51.5)	
Binge drinking in the past year			0.775
No	10,765 (48.2)	11,559 (51.8)	
Yes	1,670 (48.0)	1,812 (52.0)	
Gender			<0.001
Male	6,247 (49.5)	6,369 (50.5)	
Female	6,192 (46.9)	7,003 (53.1)	
Residential area			<0.001
Urban	6,767 (55.3)	5,463 (44.7)	
Rural	5,672 (41.8)	7,909 (58.2)	

Also, the utilization of diabetic kidney disease complication test was significantly affected by the recognition of own blood pressure level, the recognition of own blood glucose level, non-drug treatment for diabetes, oral diabetes medication, diabetes insulin treatment, diabetes management education, the completion of diabetes management education, the number of glycated hemoglobin tests in the past year, age, household composition, economic activity, education level, marital status, subjective health status, regular moderate level exercise, smoking, gender, and residential area (p<0.05).

### Predictors for the Non-Utilization of Complication Examination in South Korean Diabetic Patients

The results of logistic regression analysis for predicting the non-utilization of complication tests (kidney disease and eye disease) in South Korean diabetic patients are presented in **Tables 3**, **4**, respectively. The analysis results of the adjusted model for predicting the non-utilization of the eye disease examination in diabetic patients found independent influencing factors, which were not-knowing own blood press level (AOR=1.11, 95% CI: 1.02, 1.21), not-knowing own blood glucose level (AOR=1.23, 95% CI=1.12, 1.34), those not receiving treatment for diabetes (not taking oral diabetes medication (AOR=1.88), not receiving non-drug therapy (AOR=1.25), and not receiving insulin treatment (AOR=2.63)), non-completion of diabetes management education (AOR=1.80, 95% CI=1.65, 1.95), not conducting glycated hemoglobin tests in the past year (AOR=4.03, 95% CI=3.61, 4.51), ≤49 years old (AOR=1.26, 95% CI=1.04, 1.53), single-person household (AOR=1.14, 95% CI=1.02, 1.29), elementary school graduation or below (AOR=1.26, 95% CI=1.11, 1.42), good subjective health (AOR=1.24, 95% CI=1.12, 1.38), current smoker (AOR=1.23, 95% CI=1.11, 1.37), those who had experienced binge drinking in the past year (AOR=1.23, 95% CI=1.11, 1.37), and rural residents (AOR=1.31, 95% CI=1.22, 1.40) (p<0.05).

**Table 3:** Predictor for the non-utilization of eye disease (fundus examination) in South Korean diabetic patients: AOR and 95% CI.

Factors	AOR	95%CI	p
Recognition of own blood pressure level			
Yes (ref)	1	1	
No	1.11	1.02, 1.21	0.013
Recognition of own blood glucose level			
Yes (ref)	1	1	
No	1.23	1.12, 1.34	<0.001
Non-drug treatment for diabetes			
Yes (ref)	1	1	
No	1.25	1.17, 1.34	<0.001
Oral diabetes medication			
Yes (ref)	1	1	
No	1.88	1.61, 2.19	<0.001
Diabetes insulin treatment			
Yes (ref)	1	1	
No	2.63	2.30, 3.02	<0.001
Completion of diabetes management education			
Yes (ref)	1	1	
No	1.80	1.65, 1.95	<0.001
Number of glycated hemoglobin tests in the past year			
4 times or more (ref)	1	1	
3 times	1.16	1.05, 1.29	0.003
2 times	1.37	1.25, 1.51	<0.001
1 time	1.62	1.46, 1.80	<0.001
Not measured	4.03	3.61, 4.51	<0.001
Do not know what glycated hemoglobin is	2.65	2.43, 2.89	<0.001
Age			
≤49	1.26	1.04, 1.53	0.017
50-59	1.07	0.92, 1.24	0.366
60-69	0.89	0.78, 1.02	0.098
70-79	0.78	0.68, 0.88	<0.001
≥80(ref)	1	1	
Household composition			
Single-person household	1.14	1.02, 1.29	0.021
Two-person household	1.03	0.94, 1.13	0.452
Household with three or more members (ref)	1	1	
Economic activity			
Yes	1.14	1.05, 1.23	0.001
No (ref)	1	1	
Education level			
Elementary school graduation and below	1.26	1.11, 1.42	<0.001
Middle school graduation	1.07	0.95, 1.21	0.224
High school graduation	1.01	0.91, 1.28	0.760
College graduation or above(ref)	1	1	
Household income			
<2 million KRW	1.13	1.01, 1.26	0.030
2-3 million KRW	1.05	0.93, 1.18	0.395
3-4 million KRW	1.13	1.01, 1.28	0.034
>4 million KRW (ref)	1	1	
Subjective health status			
Good	1.24	1.12, 1.38	<0.001
Moderate	1.25	1.16, 1.35	<0.001
Poor (ref)	1	1	
Smoking			
Non-smoker (ref)	1	1	
Ex-smoker	1.05	0.97, 1.14	0.206
Current smoker	1.23	1.11, 1.37	<0.001
Binge drinking in the past year			
No (ref)	1	1	
Yes	1.23	1.11, 1.37	<0.001
Residential area			
Urban (ref)	1	1	
Rural	1.31	1.22, 1.40	<0.001

Marital status, regular moderate level exercise, and gender were excluded from the regression model by the backward selection method.

The analysis results of the adjusted model for predicting the non-utilization of the kidney disease test in diabetic patients identified independent influencing factors, which were not-knowing own blood sugar level (AOR=1.17, 95% CI=1.07, 1.28), those currently not receiving diabetes treatment (not taking oral diabetes medication (AOR=1.97), not receiving non-drug therapy (AOR=1.28), and not receiving insulin treatment (AOR=2.24)), non-completion of diabetes management education (AOR=1.61, 95% CI=1.48, 1.75), not conducting glycated hemoglobin tests in the past year (AOR=5.52, 95% CI=4.95, 6.16), single-person household (AOR=1.25, 95% CI=1.12, 1.39), good subjective health (AOR=1.26, 95% CI=1.14, 1.41), and rural residents (AOR=1.26, 95% CI=1.17, 1.36) (p<0.05).

**Table 4:** Predictors for non-utilization of kidney disease test in South Korean diabetic patients: AOR and 95% CI.

Factors	AOR	95%CI	p
Recognition of own blood pressure level			
Yes (ref)	1	1	
No	1.08	0.99, 1.18	0.060
Recognition of own blood glucose level			
Yes (ref)	1	1	
No	1.17	1.07, 1.28	<0.001
Non-drug treatment for diabetes			
Yes (ref)	1	1	
No	1.28	1.19, 1.38	<0.001
Oral diabetes medication			
Yes (ref)	1	1	
No	1.97	1.70, 2.28	<0.001
Diabetes insulin treatment			
Yes (ref)	1	1	
No	2.24	1.94, 2.59	<0.001
Completion of diabetes management education			
Yes (ref)	1	1	
No	1.61	1.48, 1.75	<0.001
Number of glycated hemoglobin tests in the past year			
4 times or more (ref)	1	1	
3 times	1.16	1.04, 1.29	0.005
2 times	1.22	1.11, 1.34	<0.001
1 time	1.57	1.41, 1.74	<0.001
Not measured	5.52	4.95, 6.16	<0.001
Do not know what glycated hemoglobin is	3.31	3.04, 3.61	<0.001
Age			
≤49	1.14	0.94, 1.37	0.174
50-59	1.01	0.87, 1.18	0.800
60-69	1.01	0.88, 1.15	0.906
70-79	0.87	0.76, 0.99	0.034
≥80(ref)	1	1	
Household composition			
Single-person household	1.25	1.12, 1.39	<0.001
Two-person household	1.10	1.01, 1.20	0.024
Household with three or more members (ref)	1	1	
Economic activity			
Yes	1.11	1.03, 1.20	0.005
No (ref)	1	1	
Subjective health status			
Good	1.26	1.14, 1.41	<0.001
Moderate	1.17	1.08, 1.26	<0.001
Poor (ref)	1	1	
Residential area			
Urban (ref)	1	1	
Rural	1.26	1.17, 1.36	<0.001

Binge drinking, gender, education level, marital status, household income, regular exercise, and smoking were excluded from the regression model by the backward selection method.

### Development and Validation of a Nomogram for Predicting the Non-Utilization of Complication Examination in South Korean Diabetic Patients

**Figure 1:**
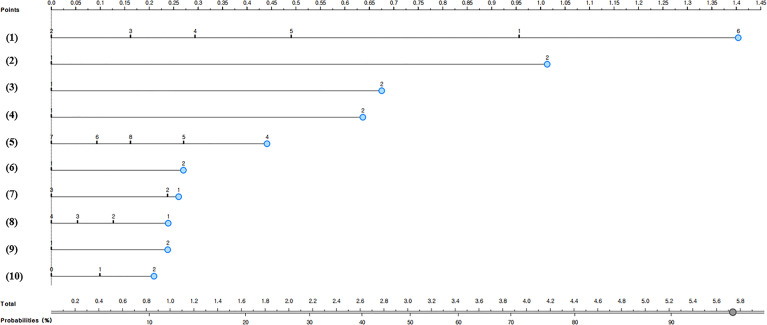
Nomogram for predicting the non-utilization of fundus examination in South Korean diabetic patients; (1) Number of glycated hemoglobin tests in the past year: 1 = don’t know what glycated hemoglobin is, 2 = 4 times or more, 3 = 3 times, 4 = 2 times, 5 = 1 time, or 6 = not measured, (2) Insulin treatment: 1=Yes or 2=No, (3) Oral diabetes medication: 1=Yes or 2=No, (4) Completion of diabetes management education: 1=Yes or 2=No, (5) Age: 4= ≤49 years old, 5 = 50-59 years old, 6 = 60-69 years old, 7 = 70-79 years old, or 8 = 80 years old or older, (6) Residential area: 1=Urban or 2=Rural, (7) Subjective health: 1 = good, 2 = moderate, or 3 = poor, (8) Education level: 1 = elementary school graduation or below, 2 = middle school graduation, 3 = high school graduation, or 4 = college graduation or above, (9) Diabetes non-drug treatment: 1 = Yes or 2 = No, and (10) Smoking: 0 = non-smoker, 1 = ex-smoker, or 2 = current smoker.

**Figure 2:**
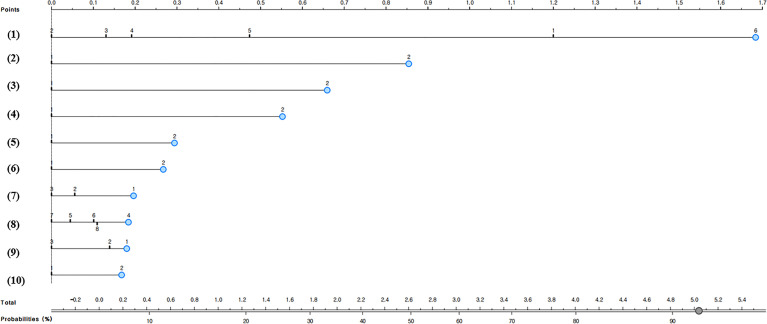
Nomogram for predicting the non-utilization of microprotein urination test in South Korean diabetic patients; (1) Number of glycated hemoglobin tests in the past year: 1 = don’t know what glycated hemoglobin is, 2 = 4 times or more, 3 = 3 times, 4 = 2 times, 5 = 1 time, or 6 = not measured, (2) Insulin treatment: 1=Yes or 2=No, (3) Oral diabetes medication: 1=Yes or 2=No, (4) Completion of diabetes management education: 1=Yes or 2=No, (5) Diabetes non-drug treatment: 1 = Yes or 2 = No, (6) Residential area: 1=Urban or 2=Rural, (7) Household composition: 1 = single-person household, 2 = two-person household, or 3 = household with three or more members, (8) Age: 4= ≤49 years old, 5 = 50-59 years old, 6 = 60-69 years old, 7 = 70-79 years old, or 8 = 80 years old or older, (9) Subjective health: 1 = good, 2 = moderate, or 3 = poor, and (10) Recognition of own blood glucose level: 1=Yes or 2=No.

**Figures 1** and **2** present the predictive nomograms for non-utilization of complication examination in South Korean diabetic patients. The nomogram for predicting the non-utilization of the fundus examination (**Figure 1**) showed that diabetic patients, who did not take the glycated hemoglobin test for the past year, did not receive diabetes treatment, did not complete diabetes management education, subjectively perceived as good in health, attended elementary school as the highest level of education, was smoking, lived in a rural area, and was 49 years or younger, had 94% chance of not utilizing the fundus examination. Moreover, the nomogram for predicting the non-utilization of the microprotein urination test (**Figure 2**) revealed that diabetic patients, who did not take the glycated hemoglobin test for the past year, did not receive diabetes treatment, did not complete diabetes management education, subjectively perceived as good in health, did not know own blood glucose level, lived in a rural area, and was 49 years or younger, had 92% chance of not utilizing the microprotein urination test.

**Figure 3:**
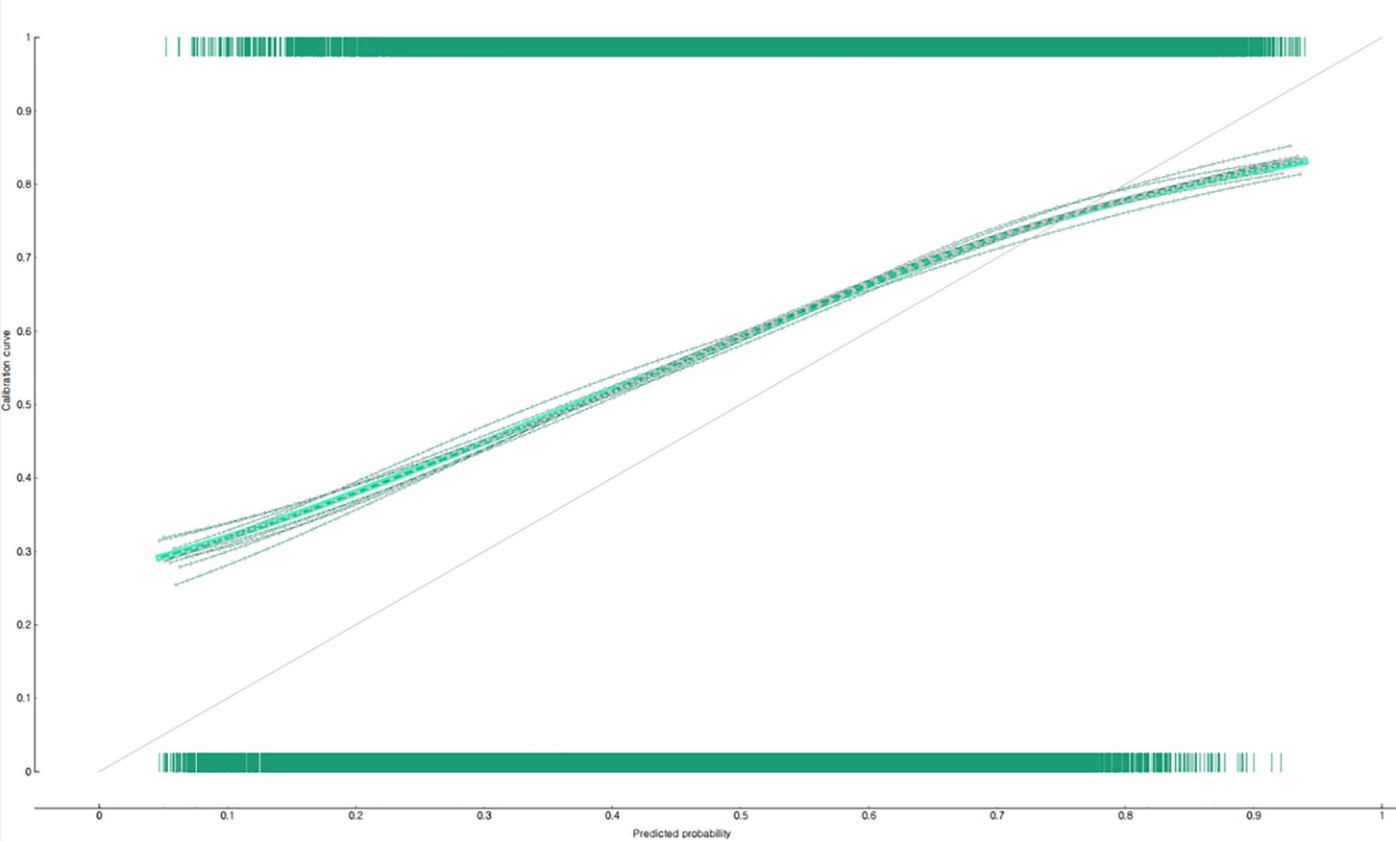
Prediction performance of the nomogram for predicting the non-utilization of fundus examination in South Korean diabetic patients: calibration plot.

**Figure 4:**
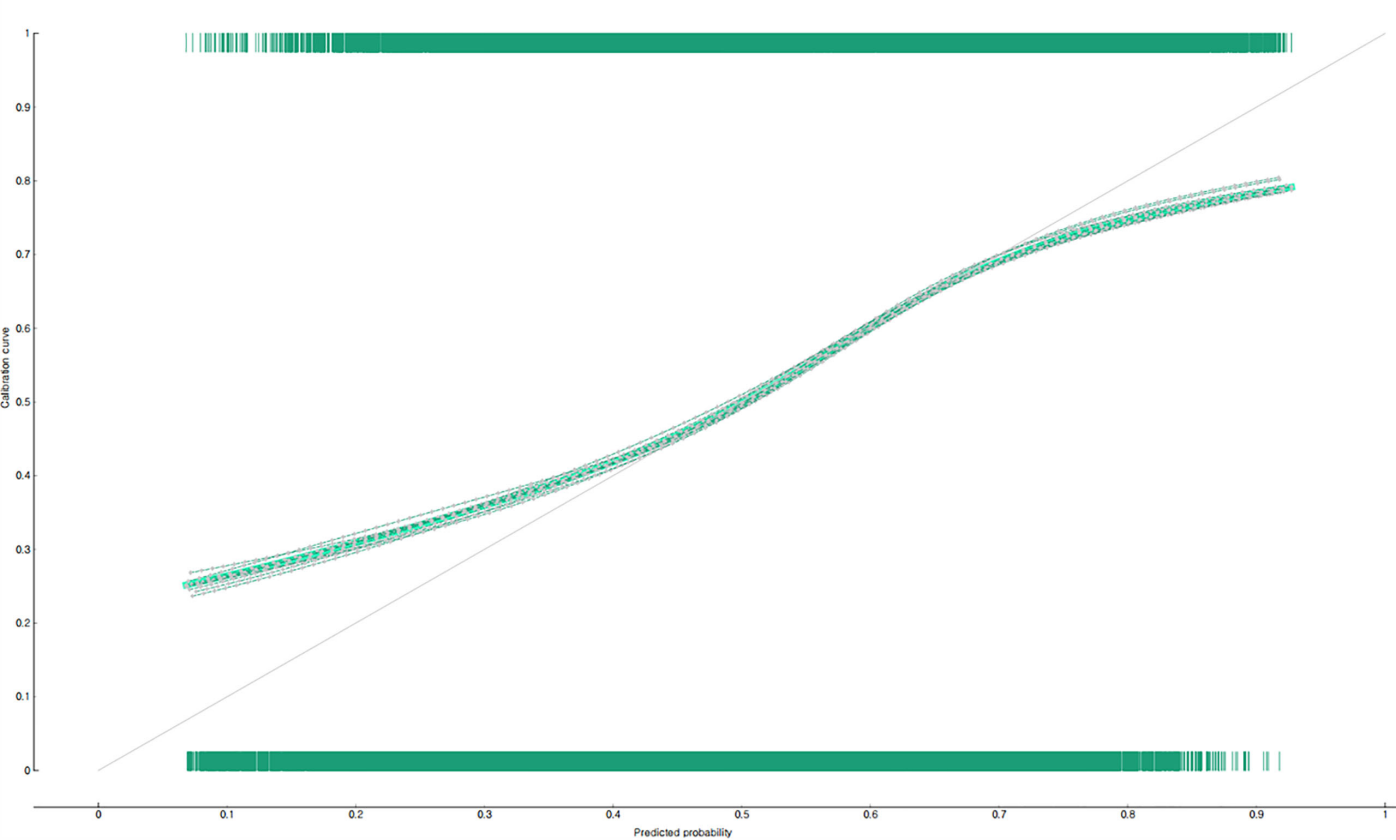
Prediction performance of the nomogram for predicting the non-utilization of microprotein urination test in South Korean diabetic patients: calibration plot.

The predictive performance of the developed two nomograms for predicting the non-utilization of diabetic complication tests was examined by using calibration plots (**Figures 3**, **4**), AUC, and accuracy. The predicted and observed probabilities were compared using the calibration plot and chi-square test for the group that received the diabetes complication tests and the group that did not receive them (**Figures 3**, **4**). Both nomograms did not show a significant difference between the predicted and observed probabilities (p<0.05). The results of 10-fold cross validation showed that the AUC, general accuracy, precision, recall, and F1-score of the nomogram for predicting the non-utilization of the fundus examination were 0.70, 0.68, 0.67, 0.70, and 0.68, respectively, and those of the nomogram for predicting the non-utilization of the microprotein urination test were 0.73, 0.69, 0.68, 0.69, and 0.69.

## Discussion

This study used the data of a national survey collected after the COVID-19 pandemic and identified factors related to the utilization of complication tests for patients with diabetes. The results of this study showed that age, education level, the recognition of own blood glucose level, current diabetes treatment, diabetes management education, the non-utilization of glycated hemoglobin test in the past year, smoking, single-person household, subjective health, and rural residents were independently related to the non-utilization of diabetes complication test. Particularly, the diabetic patients who recognized own blood glucose levels and were receiving diabetes treatment utilized fundus examination and microprotein urination test more frequently, and the group, which received diabetes management education, utilized tests for detecting diabetes complications more, which agreed with the results of previous studies (10, 11, 18, 19). Since the COVID-19 pandemic, the number of diabetic patients suffering from diabetic complications has increased, and the risk of mortality due to them is also increasing (20). Therefore, controlling the blood glucose level and receiving complication examination are important for diabetic patients for diabetic complications during the pandemic (21). It will be possible to increase the rate of utilizing the diabetes complication examination by having diabetic patients recognize blood glucose levels in the primary medical field, actively recommending diabetes treatment, conducting diabetes complication management education for diabetic patients, and monitoring blood glucose level management based on the results of this study.

The results of this study showed that the prediction probability of not utilizing the fundus examination was higher in people who were 49 years or younger than older adults, which was similar to the results of previous studies that middle-aged people utilized diabetes complication screening tests such as the microprotein urination test and the fundus examination less than older adults (11, 22). Moreover, the results of this study revealed that education level was related to the non-utilization of diabetes complication screening tests. In this study, it was predicted that diabetic patients whose highest level of education was elementary school graduation or below would utilize the fundus examination less than those whose highest level of education was college graduation or above, which agreed with Van Eijk et al. (2012) (23). Since a longer duration of diabetes increases the possibility of complication occurrence (24), it is important for middle-aged diabetic patients to actively participate in the microprotein urination test and the fundus examination to prevent diabetic complications. It is believed that customized diabetes management education targeting middle-aged diabetic patients and diabetic patients whose highest level of education was elementary school graduation or below will be needed.

The results of this study showed that diabetic patients who responded that their subjective health were moderate or poor utilized both the fundus examination and microprotein urination more than those who indicated that their subjective health were good. Previous studies also confirmed this result (10, 11, 13, 15). It could be because when people judged themselves healthier, their positive thoughts had a negative correlation with preventive health behaviors such as regular health checkups (11, 15). Therefore, it is needed that the primary medical care should provide regular notice and education about regular complication examination for diabetic patients who believe that they are in good health and monitor whether they utilize complication screening continuously.

Another finding of this study was that diabetic patients living in the rural area had a significantly higher risk of not utilizing both microprotein urination test and fundus examination than those living in the urban area. These results were consistent with the results of previous studies showing that rural residents had a lower awareness level of diabetes compared to those living in cities (25, 26) and had a higher risk of diabetic complications (27–29). Rural areas in South Korea have been losing young adults and middle-aged people and experiencing aging due to the rapidly increasing gap in socio-economic development over the past 20 years (30). Even though the demand for health and welfare is rapidly increasing, they are concerned about low accessibility to medical treatment due to the lack of medical institutions and medical professionals (30). It is believed that diabetic patients living in rural areas utilized diabetes complication tests less than those living in urban areas. Active diabetes prevention and management are necessary at the community level (e.g., diabetes complication screening tests are included in the physical examination items of the current national physical examination system) to reduce the difference in the rate of utilizing diabetic complication screening tests between regions.

Especially, this study evaluated the multiple risk factors of not utilizing complication screening tests in diabetic patients by using a nomogram. This study predicted that the probability of not utilizing the fundus examination was 94% when diabetic patients were “diabetic patients, who did not take the glycated hemoglobin test for the past year, did not receive diabetes treatment, did not complete diabetes management education, subjectively perceived as good in health, attended elementary school as the highest level of education, was smoking, lived in a rural area, and was 49 years or younger.” This study also predicted that the probability of not using the microprotein urination test was 92% when diabetic patients were “diabetic patients, who did not take the glycated hemoglobin test for the past year, did not receive diabetes treatment, did not complete diabetes management education, subjectively perceived as good in health, did not know own blood glucose level, lived in a rural area, and was 49 years or younger.” Therefore, it will be necessary to monitor the complication screening test for diabetic patients who are exposed to these multiple risk factors.

The Centers for Disease Control and Prevention, the United States of America, has monitored diabetes complications on a regular basis and operated a complication prevention program (31). On the other hand, South Korea lacks a systematic monitoring system for managing diabetic complications (32, 33). The status of diabetic complications has been studied mainly using health insurance data and hospital registry data (32, 33). Especially, complications management education for diabetic patients has been conducted in general hospitals, not primary care, in South Korea (32). Considering that general hospitals act as the emergency medical response systems in the disaster situation after the COVID-19 pandemic (34), it is needed to establish a systematic complication test and monitoring system centered on primary care. Furthermore, it is necessary to prepare systematic diabetes complications management education and customized test support policy for the subject, not top-down management centered on medical institutions, to increase the rate of utilizing diabetes complication screening tests for diabetic patients by considering health-related factors such as medically vulnerable areas and diabetes management education, in addition to the individual characteristics of diabetic patients.

The importance of this study was to identify diabetic groups with a high risk of not utilizing complication screening tests (fundus examination and microprotein urination test) by using a national survey data representing South Korean communities and provide evidence for effectively preventing complications in diabetic patients. This study had several limitations. First, this study had a probability of underestimation or overestimation due to the recall bias of the survey. It is because this study used face-to-face questionnaires for the utilization of the fundus examination and microprotein urination test. Therefore, future studies need to reduce the probability of recall bias by including a medical record in addition to questionnaires to accurately determine the utilization of the complication screening test. Second, the predictive model does not include information about the duration of diabetes because the Community Health Survey, the data source of this study, did not investigate it. Future studies need to evaluate the duration of diabetes to improve the predictive performance of a model for predicting the non-utilization of complication screening tests in diabetic patients. Third, in this study, we did not reflect the impact of COVID-19 on the testing of complications in patients with diabetes in their analysis. Fourth, since it was a cross-sectional study, even if factors related to the non-utilization of diabetes complication tests were identified in this study, it cannot be interpreted as a causal relationship according to temporal precedence.

## Conclusions

This study confirmed that age, education level, the recognition of own blood glucose level, current diabetes treatment, diabetes management education, not conducting the glycated hemoglobin test in the past year, smoking, single-person household, subjectively good health, and living in the rural area were independently related to the non-utilization of diabetes complication test after the COVID-19 pandemic. Therefore, it is needed to detect groups that are highly likely not to utilize diabetes complication screening tests by considering these multiple risk factors. It is also necessary to devise a customized support system at the primary care level that can increase the rate of utilizing them while considering these factors. It is required to prepare a system that can systematically monitor groups highly vulnerable to diabetic complications in medically underprivileged areas such as rural areas. Furthermore, since diabetes complication screening tests and blood sugar management are important elements in diabetes management, it is necessary to develop customized diabetes management guidelines that consider the characteristics of those who have utilized diabetes complication screening tests.

## Data Availability Statement

The raw data supporting the conclusions of this article will be made available by the authors, without undue reservation.

## Ethics Statement 

The studies involving human participants were reviewed and approved by Korea Disease Control and Prevention. The patients/participants provided their written informed consent to participate in this study.

## Author Contributions

The author confirms being the sole contributor of this work and has approved it for publication.

## Funding

This research was supported by Basic Science Research Program through the National Research Foundation of Korea (NRF) funded by the Ministry of Education (NRF- 2018R1D1A1B07041091, 2021S1A5A8062526) and 2022 Development of Open-Lab based on 4P in the Southeast Zone.

## Conflict of Interest

The author declares that the research was conducted in the absence of any commercial or financial relationships that could be construed as a potential conflict of interest.

## Publisher’s Note

All claims expressed in this article are solely those of the authors and do not necessarily represent those of their affiliated organizations, or those of the publisher, the editors and the reviewers. Any product that may be evaluated in this article, or claim that may be made by its manufacturer, is not guaranteed or endorsed by the publisher.
