# Supplementary material for: Factors Influencing the Utilization of Diabetes Complication Tests Under the COVID-19 Pandemic: Machine Learning Approach
Source: Front Endocrinol (Lausanne). 2022 Jun 22;13:925844. doi: 10.3389/fendo.2022.925844 (PMC9258727; doi:10.3389/fendo.2022.925844)
Supplement: Supplementary file 1 [file DataSheet_1.docx]

Supplementary Material

# Supplementary Tables

**Table 1**. Characteristics of subjects according to the diabetic eye complication test (fundoscopic examination), n (%)

| **Factors** | **Diabetic eye complication test** | | **p** |
| --- | --- | --- | --- |
|  | **Yes (n=10,480)** | **No (n=15,331)** |  |
| Recognition of own blood pressure level |  |  | <0.001 |
| Yes | 7,467 (44.7) | 9,233 (55.3) |  |
| No | 3,002 (33.1) | 6,057 (66.9) |  |
| Recognition of own blood glucose level |  |  | <0.001 |
| Yes | 7,700 (45.5) | 9,224 (54.5) |  |
| No | 2,765 (31.3) | 6,067 (68.7) |  |
| Non-drug treatment for diabetes |  |  | <0.001 |
| Yes | 4,108 (48.8) | 4,318 (51.2) |  |
| No | 6,368 (36.6) | 11,012 (63.4) |  |
| Oral diabetes medication |  |  | <0.001 |
| Yes | 9,995 (41.5) | 14,115 (58.5) |  |
| No | 481 (28.4) | 1,214 (71.6) |  |
| Diabetes insulin treatment |  |  | <0.001 |
| Yes | 1,294 (68.8) | 587 (31.2) |  |
| No | 9,185 (38.4) | 14,743 (61.6) |  |
| Completion of diabetes management education |  |  | <0.001 |
| Yes | 3,072 (59.7) | 2,071 (40.3) |  |
| No | 7,405 (35.8) | 13,255 (64.2) |  |
| Number of glycated hemoglobin tests in the past year |  |  | <0.001 |
| Do not know what glycated hemoglobin is | 1,942 (27.7) | 5,075 (72.3) |  |
| 4 times or more | 3,632 (57.8) | 2,653 (42.2) |  |
| 3 times | 1,327 (51.9) | 1,232 (48.1) |  |
| 2 times | 1,776 (47.5) | 1,962 (52.5) |  |
| 1 time | 1,103 (41.0) | 1,586 (59.0) |  |
| Not measured | 654 (19.3) | 2,728 (80.7) |  |
| Age |  |  | <0.001 |
| ≤49 | 609 (40.0) | 913 (60.0) |  |
| 50-59 | 1,910 (42.2) | 2,612 (57.8) |  |
| 60-69 | 3,445 (43.0) | 4,573 (57.0) |  |
| 70-79 | 3,389 (40.8) | 4,908 (59.2) |  |
| ≥80 | 1,127 (32.6) | 2,325 (67.4) |  |
| Household composition |  |  | <0.001 |
| Single-person household | 2,020 (36.3) | 3,546 (63.7) |  |
| Two-person household | 5,561 (41.0) | 7,998 (59.0) |  |
| Household with three or more members | 2,899 (43.4) | 3,787 (56.6) |  |
| Economic activity |  |  | <0.001 |
| Yes | 4,889 (38.7) | 7,757 (61.3) |  |
| No | 5,583 (42.4) | 7,569 (57.6) |  |
| Education level |  |  | <0.001 |
| Elementary school graduation and below | 3,911 (35.2) | 7,185 (64.8) |  |
| Middle school graduation | 1,906 (41.0) | 2,739 (59.0) |  |
| High school graduation | 2,949 (45.1) | 3,588 (54.9) |  |
| College graduation or above | 1,706 (48.7) | 1,799 (51.3) |  |
| Marital status |  |  | <0.001 |
| Married | 7,525 (41.9) | 10,419 (58.1) |  |
| Single | 230 (39.7) | 350 (60.3) |  |
| Divorced, widowed, or separated | 2,722 (37.4) | 4,548 (62.6) |  |
| Household income |  |  | <0.001 |
| <2 million KRW | 4,308 (37.6) | 7,159 (62.4) |  |
| 2-3 million KRW | 1,517 (43.2) | 1,991 (56.8) |  |
| 3-4 million KRW | 1,074 (43.0) | 1,424 (57.0) |  |
| >4 million KRW | 2,067 (46.4) | 2,392 (53.6) |  |
| Subjective health status |  |  | <0.001 |
| Good | 1,371 (38.1) | 2,230 (61.9) |  |
| Moderate | 3,923 (38.7) | 6,220 (61.3) |  |
| Poor | 5,186 (43.0) | 6,879 (57.0) |  |
| Regular moderate level exercise |  |  | 0.315 |
| No | 8,983 (40.5) | 13,206 (59.5) |  |
| Yes | 1,491 (41.4) | 2,113 (58.6) |  |
| Smoking |  |  | <0.001 |
| Non-smoker | 5,979 (41.2) | 8,530 (58.8) |  |
| Ex-smoker | 3,040 (41.5) | 4,287 (58.5) |  |
| Current smoker | 1,461 (36.8) | 2,514 (63.2) |  |
| Binge drinking in the past year |  |  | <0.001 |
| No | 9,205 (41.2) | 13,119 (58.8) |  |
| Yes | 1,273 (36.6) | 2,209 (63.4) |  |
| Gender |  |  | 0.317 |
| Male | 5,083 (40.3) | 7,533 (59.7) |  |
| Female | 5,397 (40.9) | 7,798 (59.1) |  |
| Residential area |  |  | <0.001 |
| Urban | 5,772 (47.2) | 6,458 (52.8) |  |
| Rural | 4,708 (34.7) | 8,873 (65.3) |  |

**Table 2.** Characteristics of subjects according to the diabetic kidney complication test (microprotein urination test), n (%)

| **Factors** | **Diabetic kidney complication test** | | **p** |
| --- | --- | --- | --- |
|  | **Yes (n=12,439)** | **No (n=13,372)** |  |
| Recognition of own blood pressure level |  |  | <0.001 |
| Yes | 8,801 (52.7) | 7,899 (47.3) |  |
| No | 3,621 (40.0) | 5,438 (60.0) |  |
| Recognition of own blood glucose level |  |  | <0.001 |
| Yes | 9,058 (53.5) | 7,866 (46.5) |  |
| No | 3,362 (38.1) | 5,470 (61.9) |  |
| Non-drug treatment for diabetes |  |  | <0.001 |
| Yes | 4,830 (57.3) | 3,596 (42.7) |  |
| No | 7,608 (43.8) | 9,772 (56.2) |  |
| Oral diabetes medication |  |  | <0.001 |
| Yes | 11,865 (49.2) | 12,245 (50.8) |  |
| No | 571 (33.7) | 1,124 (66.3) |  |
| Diabetes insulin treatment |  |  | <0.001 |
| Yes | 1,364 (72.5) | 517 (27.5) |  |
| No | 11,074 (46.3) | 12,854 (53.7) |  |
| Completion of diabetes management education |  |  | <0.001 |
| Yes | 3,409 (66.3) | 1,734 (33.7) |  |
| No | 9,027 (43.7) | 11,633 (56.3) |  |
| Number of glycated hemoglobin tests in the past year |  |  | <0.001 |
| Do not know what glycated hemoglobin is | 2,200 (31.4) | 4,817 (68.6) |  |
| 4 times or more | 4,207 (66.9) | 2,078 (33.1) |  |
| 3 times | 1,603 (62.6) | 956 (37.4) |  |
| 2 times | 2,250 (60.2) | 1,488 (39.8) |  |
| 1 time | 1,391 (51.7) | 1,298 (48.3) |  |
| Not measured | 732 (21.6) | 2,650 (78.4) |  |
| Age |  |  | <0.001 |
| ≤49 | 798 (52.4) | 724 (47.6) |  |
| 50-59 | 2,446 (54.1) | 2,076 (45.9) |  |
| 60-69 | 3,979 (49.6) | 4,039 (50.4) |  |
| 70-79 | 3,886 (46.8) | 4,411 (53.2) |  |
| ≥80 | 1,330 (38.5) | 2,122 (61.5) |  |
| Household composition |  |  | <0.001 |
| Single-person household | 2,328 (41.8) | 3,238 (58.2) |  |
| Two-person household | 6,567 (48.4) | 6,992 (51.6) |  |
| Household with three or more members | 3,544 (53.0) | 3,142 (47.0) |  |
| Economic activity |  |  | 0.002 |
| Yes | 5,968 (47.2) | 6,678 (52.8) |  |
| No | 6,463 (49.1) | 6,689 (50.9) |  |
| Education level |  |  | <0.001 |
| Elementary school graduation and below | 4,623 (41.7) | 6,473 (58.3) |  |
| Middle school graduation | 2,258 (48.6) | 2,387 (51.4) |  |
| High school graduation | 3,535 (54.1) | 3,002 (45.9) |  |
| College graduation or above | 2,009 (57.3) | 1,496 (42.7) |  |
| Marital status |  |  | <0.001 |
| Married | 9,010 (50.2) | 8,934 (49.8) |  |
| Single | 261 (45.0) | 319 (55.0) |  |
| Divorced, widowed, or separated | 3,160 (43.5) | 4,110 (56.5) |  |
| Household income |  |  | <0.001 |
| <2 million KRW | 5,031 (43.9) | 6,436 (56.1) |  |
| 2-3 million KRW | 1,793 (51.1) | 1,715 (48.9) |  |
| 3-4 million KRW | 1,348 (54.0) | 1,150 (46.0) |  |
| >4 million KRW | 2,483 (55.7) | 1,976 (44.3) |  |
| Subjective health status |  |  | 0.006 |
| Good | 1,667 (46.3) | 1,934 (53.7) |  |
| Moderate | 4,844 (47.8) | 5,299 (52.2) |  |
| Poor | 5,928 (49.1) | 6,137 (50.9) |  |
| Regular moderate level exercise |  |  | 0.003 |
| No | 10,607 (47.8) | 11,582 (52.2) |  |
| Yes | 1,820 (50.5) | 1,784 (49.5) |  |
| Smoking |  |  | 0.022 |
| Non-smoker | 6,890 (47.5) | 7,619 (52.5) |  |
| Ex-smoker | 3,623 (49.4) | 3,704 (50.6) |  |
| Current smoker | 1,926 (48.5) | 2,049 (51.5) |  |
| Binge drinking in the past year |  |  | 0.775 |
| No | 10,765 (48.2) | 11,559 (51.8) |  |
| Yes | 1,670 (48.0) | 1,812 (52.0) |  |
| Gender |  |  | <0.001 |
| Male | 6,247 (49.5) | 6,369 (50.5) |  |
| Female | 6,192 (46.9) | 7,003 (53.1) |  |
| Residential area |  |  | <0.001 |
| Urban | 6,767 (55.3) | 5,463 (44.7) |  |
| Rural | 5,672 (41.8) | 7,909 (58.2) |  |

**Table 3.** Predictor for the non-utilization of eye disease (fundus examination) in South Korean diabetic patients: AOR and 95% CI

| **Factors** | **AOR** | **95%CI** | **p** |
| --- | --- | --- | --- |
| Recognition of own blood pressure level |  |  |  |
| Yes (ref) | 1 | 1 |  |
| No | 1.11 | 1.02, 1.21 | 0.013 |
| Recognition of own blood glucose level |  |  |  |
| Yes (ref) | 1 | 1 |  |
| No | 1.23 | 1.12, 1.34 | <0.001 |
| Non-drug treatment for diabetes |  |  |  |
| Yes (ref) | 1 | 1 |  |
| No | 1.25 | 1.17, 1.34 | <0.001 |
| Oral diabetes medication |  |  |  |
| Yes (ref) | 1 | 1 |  |
| No | 1.88 | 1.61, 2.19 | <0.001 |
| Diabetes insulin treatment |  |  |  |
| Yes (ref) | 1 | 1 |  |
| No | 2.63 | 2.30, 3.02 | <0.001 |
| Completion of diabetes management education |  |  |  |
| Yes (ref) | 1 | 1 |  |
| No | 1.80 | 1.65, 1.95 | <0.001 |
| Number of glycated hemoglobin tests in the past year |  |  |  |
| 4 times or more (ref) | 1 | 1 |  |
| 3 times | 1.16 | 1.05, 1.29 | 0.003 |
| 2 times | 1.37 | 1.25, 1.51 | <0.001 |
| 1 time | 1.62 | 1.46, 1.80 | <0.001 |
| Not measured | 4.03 | 3.61, 4.51 | <0.001 |
| Do not know what glycated hemoglobin is | 2.65 | 2.43, 2.89 | <0.001 |
| Age |  |  |  |
| ≤49 | 1.26 | 1.04, 1.53 | 0.017 |
| 50-59 | 1.07 | 0.92, 1.24 | 0.366 |
| 60-69 | 0.89 | 0.78, 1.02 | 0.098 |
| 70-79 | 0.78 | 0.68, 0.88 | <0.001 |
| ≥80(ref) | 1 | 1 |  |
| Household composition |  |  |  |
| Single-person household | 1.14 | 1.02, 1.29 | 0.021 |
| Two-person household | 1.03 | 0.94, 1.13 | 0.452 |
| Household with three or more members (ref) | 1 | 1 |  |
| Economic activity |  |  |  |
| Yes | 1.14 | 1.05, 1.23 | 0.001 |
| No (ref) | 1 | 1 |  |
| Education level |  |  |  |
| Elementary school graduation and below | 1.26 | 1.11, 1.42 | <0.001 |
| Middle school graduation | 1.07 | 0.95, 1.21 | 0.224 |
| High school graduation | 1.01 | 0.91, 1.28 | 0.760 |
| College graduation or above(ref) | 1 | 1 |  |
| Household income |  |  |  |
| <2 million KRW | 1.13 | 1.01, 1.26 | 0.030 |
| 2-3 million KRW | 1.05 | 0.93, 1.18 | 0.395 |
| 3-4 million KRW | 1.13 | 1.01, 1.28 | 0.034 |
| >4 million KRW (ref) | 1 | 1 |  |
| Subjective health status |  |  |  |
| Good | 1.24 | 1.12, 1.38 | <0.001 |
| Moderate | 1.25 | 1.16, 1.35 | <0.001 |
| Poor (ref) | 1 | 1 |  |
| Smoking |  |  |  |
| Non-smoker (ref) | 1 | 1 |  |
| Ex-smoker | 1.05 | 0.97, 1.14 | 0.206 |
| Current smoker | 1.23 | 1.11, 1.37 | <0.001 |
| Binge drinking in the past year |  |  |  |
| No (ref) | 1 | 1 |  |
| Yes | 1.23 | 1.11, 1.37 | <0.001 |
| Residential area |  |  |  |
| Urban (ref) | 1 | 1 |  |
| Rural | 1.31 | 1.22, 1.40 | <0.001 |

*Marital status, regular moderate level exercise, and gender were excluded from the regression model by the backward selection method.

**Table 4.** Predictors for non-utilization of kidney disease test in South Korean diabetic patients: AOR and 95% CI

| **Factors** | **AOR** | **95%CI** | **p** |
| --- | --- | --- | --- |
| Recognition of own blood pressure level |  |  |  |
| Yes (ref) | 1 | 1 |  |
| No | 1.08 | 0.99, 1.18 | 0.060 |
| Recognition of own blood glucose level |  |  |  |
| Yes (ref) | 1 | 1 |  |
| No | 1.17 | 1.07, 1.28 | <0.001 |
| Non-drug treatment for diabetes |  |  |  |
| Yes (ref) | 1 | 1 |  |
| No | 1.28 | 1.19, 1.38 | <0.001 |
| Oral diabetes medication |  |  |  |
| Yes (ref) | 1 | 1 |  |
| No | 1.97 | 1.70, 2.28 | <0.001 |
| Diabetes insulin treatment |  |  |  |
| Yes (ref) | 1 | 1 |  |
| No | 2.24 | 1.94, 2.59 | <0.001 |
| Completion of diabetes management education |  |  |  |
| Yes (ref) | 1 | 1 |  |
| No | 1.61 | 1.48, 1.75 | <0.001 |
| Number of glycated hemoglobin tests in the past year |  |  |  |
| 4 times or more (ref) | 1 | 1 |  |
| 3 times | 1.16 | 1.04, 1.29 | 0.005 |
| 2 times | 1.22 | 1.11, 1.34 | <0.001 |
| 1 time | 1.57 | 1.41, 1.74 | <0.001 |
| Not measured | 5.52 | 4.95, 6.16 | <0.001 |
| Do not know what glycated hemoglobin is | 3.31 | 3.04, 3.61 | <0.001 |
| Age |  |  |  |
| ≤49 | 1.14 | 0.94, 1.37 | 0.174 |
| 50-59 | 1.01 | 0.87, 1.18 | 0.800 |
| 60-69 | 1.01 | 0.88, 1.15 | 0.906 |
| 70-79 | 0.87 | 0.76, 0.99 | 0.034 |
| ≥80(ref) | 1 | 1 |  |
| Household composition |  |  |  |
| Single-person household | 1.25 | 1.12, 1.39 | <0.001 |
| Two-person household | 1.10 | 1.01, 1.20 | 0.024 |
| Household with three or more members (ref) | 1 | 1 |  |
| Economic activity |  |  |  |
| Yes | 1.11 | 1.03, 1.20 | 0.005 |
| No (ref) | 1 | 1 |  |
| Subjective health status |  |  |  |
| Good | 1.26 | 1.14, 1.41 | <0.001 |
| Moderate | 1.17 | 1.08, 1.26 | <0.001 |
| Poor (ref) | 1 | 1 |  |
| Residential area |  |  |  |
| Urban (ref) | 1 | 1 |  |
| Rural | 1.26 | 1.17, 1.36 | <0.001 |

*Binge drinking, gender, education level, marital status, household income, regular exercise, and smoking were excluded from the regression model by the backward selection method.
